# Sarcomatoid Mesothelioma With New Pancreatic Lesions Presenting As Acute Pancreatitis: A Case Report

**DOI:** 10.7759/cureus.64088

**Published:** 2024-07-08

**Authors:** Feras Al-Moussally, Faris Alamin, Saud Khan, Priya K Gopalan

**Affiliations:** 1 Internal Medicine, University of Central Florida College of Medicine, Kissimmee, USA; 2 Internal Medicine, University of Central Florida-HCA Osceola Hospital, Orlando, USA; 3 Hematology/Oncology, Orlando VA Medical Center, Orlando, USA

**Keywords:** metastasis, mesothelioma, sarcomatoid, acute, pancreatitis

## Abstract

Sarcomatoid mesothelioma is a rare, aggressive malignancy that usually follows asbestos exposure. It is the least common subtype of mesotheliomas, following epithelial and biphasic subtypes. Pleural mesothelioma can metastasize, with the liver, kidneys, adrenal glands, and opposite lungs being the most commonly reported sites for metastasis. Metastasis of the pancreas is extremely rare, which is why the authors of this case report intend to present the case of a 78-year-old male who was found to have acute pancreatitis, most likely secondary to metastatic lesions.

## Introduction

Sarcomatoid cells are typically present in the bones, nerves, and connective tissues. Sarcomatoid mesothelioma is the rarest subtype of malignant mesothelioma with a median survival of four months with surgical treatment and 15 months with immunotherapy. It usually occurs following exposure to asbestos and is characterized by spindle cell proliferation under microscopy. Pleural mesothelioma most often metastasizes to the liver, spleen, kidneys, and adrenal glands. We intend to present a case of a 78-year-old male with a history of pleural sarcomatoid mesothelioma who presented to the emergency department with abdominal pain and was found to have acute pancreatitis likely secondary to metastatic lesions found in the neck and tail of the pancreas.

## Case presentation

**Figure 1:**
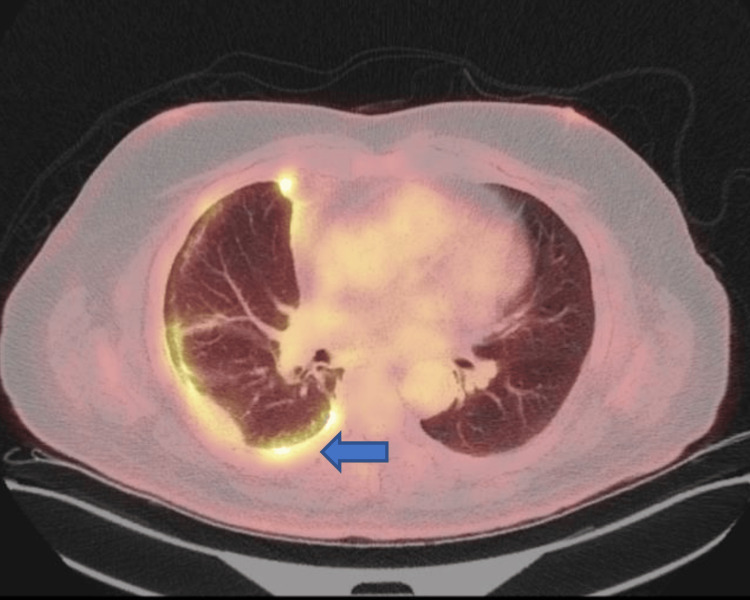
PET scan showing increased FDG activity in multiple foci throughout the right pleura, mostly posteriorly (arrow) PET: positron emission tomography; FDG: fluorodeoxyglucose

The patient is a 78-year-old male with a past medical history of diabetes mellitus, hyperlipidemia, benign prostatic hypertrophy, hypertension, allergic rhinitis, and remote history of possible occupational asbestos exposure, who was recently diagnosed with metastatic sarcomatoid mesothelioma (Figure 1), who came to the emergency department complaining of a three-day history of abdominal pain, nausea, and vomiting.

The patient reported that he had progressively worsening food intolerance with epigastric abdominal discomfort. He had multiple non-bloody and nonbilious vomiting episodes the day prior to the presentation. At home, the patient was taking acetaminophen 325mg three times a day, an albuterol inhaler every six hours, amlodipine 10mg once a day, docusate/sennosides 50mg/8.6mg once a day, fluticasone/salmeterol 250/50 twice a day, glipizide 5mg once a day, losartan 100mg once a day, metoprolol tartrate 25mg twice a day, oxycodone 10mg three times a day, pantoprazole 40mg once a day, and rosuvastatin 40mg once a day.

Notably, seven weeks prior to presentation, the patient underwent a biopsy of a chest wall mass which was consistent with a malignant spindle cell neoplasm, favoring sarcomatoid mesothelioma. Immunohistochemistry was positive for AE1/AE3, Cam52, OSCAR, D2-40, calretinin (weak and patchy), actin and GATA3; nonspecific for CD31, SATB2; negative for WT-1, ERG, CD34, STAT6, CK7, NKX3.1, Sox10, S100, SMMS-1, MyoD1, myogenin, CDK4, MDM2, TTF-1, p40 and Pax8; H3-trimethyl K27 nuclear expression preserved. Subsequently, he underwent right thoracotomy with resection of the chest wall mass and received palliative radiation therapy with the last session being a month prior to the presentation described.

In the emergency department, the patient was afebrile, pulse of 91 beats per minute, respiratory rate of 18 breaths per minute, and blood pressure of 158/80 mmHg. His laboratory investigation was significant for the following values in Table 1.

**Table 1:** Pertinent laboratory findings

Laboratory test	Value	Reference range
Lipase	>1020 U/L	8-78 U/L
White blood cell	12.1 K/cmm	4.2-10.3 K/cmm
Aspartate aminotransferase	12 U/L	5-34 U/L
Alanine aminotransferase	12 UL	10-55 U/L
Total bilirubin	0.38 mg/dL	0.16-1.25 mg/dL

**Figure 2:**
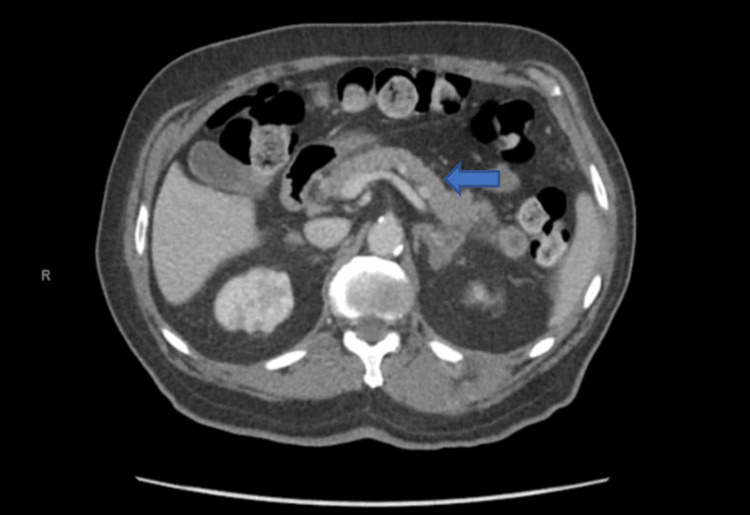
CT scan of the abdomen showing multiple new pancreatic metastases (arrow)

The patient had a computed tomography (CT) of the abdomen and pelvis which showed possible mild pancreatitis associated with new masses in the pancreatic neck and tail (Figure 2). The patient also had worsening metastatic disease involving the peritoneum, paraspinal muscle, and osseous involvement.

The patient was admitted to the hospital for the management of acute pancreatitis. He was initially placed on no diet and slowly progressed as tolerated, started on intravenous fluid replacement, and a pain control regimen. The patient slowly improved and was discharged home with plans to follow up with his oncologist to start the planned regimen of nivolumab and ipilimumab once acute pancreatitis resolved.

## Discussion

Mesothelial tumors are divided into benign or preinvasive and mesotheliomas. The benign subgroup includes mesothelioma in situ, well-differentiated papillary mesothelial tumors, and adenomatoid tumors. Invasive mesothelioma is divided histologically into epithelial, biphasic, and sarcomatoid [1]. Epithelial subtype is the most common invasive mesothelioma subtype comprising 60% of cases, followed by biphasic which comprises 20%, and lastly, the sarcomatoid subtype is the rarest [2]. Identifying the subtype of mesothelioma is important since it impacts treatment plans and prognosis discussions [3]. The median survival in patients diagnosed with sarcomatoid mesothelioma who undergo surgical treatment is 4 months, compared to 19 months and 12 months in epithelioid and biphasic subtypes [3]. In a recent study, it was shown that the median survival for sarcomatoid mesothelioma could reach as high as 15 months with immunotherapy [2]. The worse prognosis of the sarcomatoid subtype is thought to be due to the subtype’s ability to invade the surrounding tissue, rapid growth, inconsistent expression of tumor markers, and fibrous nature [2,4].

The primary cause of sarcomatoid mesothelioma is thought to be asbestos exposure [2]. Cancer can arise after 20 years to 60 years following exposure [2]. Presenting symptoms vary but could include fatigue, anemia, anorexia, and similar to our patient, chest pain, cough, and hemoptysis [2]. It is defined as spindle cell proliferation in fascicles or haphazard patterns invading the lung parenchyma or adipose tissue [1]. Investigation studies include CDKN2A, MTAP, BAP1, cytokeratins, mesothelial markers, FLI1, CD31, ERG, CD34, STAT6, myogenin, S-100 protein, melan A, HMB45, SOX10 [1]. Pleural mesothelioma has the ability to metastasize. In fact, a study in 2012 showed more than half the patients had distant metastases with the most common site being liver, followed by adrenal glands, kidneys, and the opposite lung [2]. However, metastasis to the pancreas is extremely rare [5].

There is a chance of diagnostic inaccuracy for mesotheliomas as there are multiple dyes useful for immunohistochemistry, the combination of which is unique for different mesotheliomas, and the interpretation of these studies is subjective. Thus, it is recommended that a team of pathologists with proven experience in diagnosing mesothelioma confirm the diagnosis in cases of diagnostic uncertainty [6-7].

## Conclusions

Our patient improved after the management of pancreatitis per the guidelines. He was later discharged and was planned to start immunotherapy outpatient. The authors of this case report intended to present a case highlighting the pancreas as a potential metastasis location for pleural sarcomatoid mesothelioma. In addition, metastatic disease involving the pancreas is an important differential diagnosis for acute pancreatitis.
